# USP54 Promotes Ferroptosis in Non‐Small Cell Lung Cancer by Mediating FOXA2 Deubiquitination and Enhancing ACSL4 Transcription

**DOI:** 10.1002/kjm2.70139

**Published:** 2025-11-20

**Authors:** Rui‐Shi Wei, Yong‐Ping Liu, Chun‐Dong Gu, Shi‐Lei Zhao, Li Feng, Jian‐Rong Yu

**Affiliations:** ^1^ Department of Thoracic Surgery Changzhou Cancer Hospital Changzhou Jiangsu China; ^2^ Department of Oncology Changzhou Cancer Hospital Changzhou Jiangsu China; ^3^ Department of Thoracic Surgery The First Affiliated Hospital of Dalian Medical University Dalian Liaoning China

**Keywords:** ACSL4, ferroptosis, FOXA2, non‐small cell lung cancer, USP54

## Abstract

Non‐small cell lung cancer (NSCLC) is the most common type of lung cancer, with a 5‐year survival rate of less than 20% and a high risk of recurrence despite advances in treatment. This study aimed to identify new therapeutic targets to increase the effectiveness of NSCLC treatments. We examined the role of USP54 in ferroptosis using an MTT assay and assessed the levels of reactive oxygen species (ROS), ferrous iron (Fe^2+^), and malondialdehyde (MDA). To explore the underlying molecular mechanism, the intermolecular interactions was assessed using coimmunoprecipitation (Co‐IP), chromatin immunoprecipitation (ChIP), and dual‐luciferase reporter assays. We found that USP54 expression was reduced in NSCLC and that its overexpression inhibited NSCLC cell proliferation while inducing ferroptosis, as indicated by increased ROS, Fe^2+^, and MDA levels, along with changes in SLC7A11, GPX4, and ACSL4 expression. Additionally, USP54 mediated the deubiquitination of FOXA2, decreasing its degradation. And FOXA2 promoted ACSL4 transcription, which further induced ferroptosis in NSCLC cells. In conclusion, USP54 promotes ferroptosis and inhibits NSCLC progression by stabilizing FOXA2, which in turn activates ACSL4 transcription. This study provides a theoretical foundation for the development of therapies targeting USP54 or ACSL4 for NSCLC treatment.

**Abbreviations**
ACSL4: acyl‐CoA synthetase long‐chain family member 4
ANOVA: analysis of variance
BCA: bicinchoninic acid
ChIP: chromatin immunoprecipitation
CoIP: coimmunoprecipitation
DUBs: deubiquitinases FBS—fetal bovine serum
Fer‐1: ferrostatin‐1 (ferroptosis inhibitor)
FOXA2: forkhead box A2
GAPDH: glyceraldehyde‐3‐phosphate dehydrogenase
GEPIA: gene expression profiling interactive analysis
HIF2α: hypoxia‐inducible factor 2 alpha
IHC: immunohistochemistry
JASPAR: database for transcription factor binding profiles
MDA: malondialdehyde
MTT: 3‐(4,5‐dimethylthiazol‐2‐yl)‐2,5‐diphenyltetrazolium bromide
NCBI: national center for biotechnology information
NSCLC: non‐small cell lung cancer PVDF—Polyvinylidene fluoride
qPCR: quantitative polymerase chain reaction
qPCR: reverse transcription quantitative PCR
RIP: RNA immunoprecipitation
RIPA: radioimmunoprecipitation assay
ROS: reactive oxygen species
SDS–PAGE: sodium dodecyl sulfate‐polyacrylamide gel electrophoresis
SLC7A11: solute carrier family 7 member 11
USP54: ubiquitin‐specific protease 54

## 1 Introduction

Lung cancer is one of the most lethal cancers worldwide, with non‐small cell lung cancer (NSCLC) accounting for approximately 80%–85% of all cases [1]. It has the highest incidence and mortality rates among common malignancies, making it a major public health concern in China [2]. Currently, the diagnosis of lung cancer relies primarily on imaging techniques, which result in many patients being diagnosed at advanced stages [2]. The treatment of NSCLC mainly includes surgery, radiotherapy, chemotherapy, and immunotherapy. However, clinical outcomes remain poor, largely due to the aggressive nature of the disease and the influence of patients' general health and socioeconomic status on treatment accessibility and effectiveness [3, 4]. Therefore, there is an urgent need to identify novel therapeutic targets and develop more effective treatment options for lung cancer.

Ferroptosis, a recently identified form of regulated cell death, has demonstrated anticancer effects in various tumor models and has attracted significant attention both domestically and internationally for its potential as a therapeutic strategy [5]. Acyl‐CoA synthetase long chain family member 4 (ACSL4) is an enzyme that catalyzes the conversion of fatty acids into acyl‐CoA esters, playing a crucial role in lipid biosynthesis and metabolism regulation [6]. Existing research has shown that ACSL4 is essential for the execution of ferroptosis and is widely recognized as a key mediator of this process [7, 8]. Furthermore, in NSCLC progression, ACSL4 plays a significant role by regulating ferroptosis. For instance, circSCN8A can induce ferroptosis by modulating the miR‐1290/ACSL4 axis, thereby inhibiting NSCLC cell proliferation and metastasis, suggesting that ACSL4 may function as a tumor suppressor in NSCLC development [9]. Forkhead box A2 (FOXA2), a member of the FOXA transcription factor family, is a critical regulator of embryonic development [10]. In recent years, growing evidence has highlighted its important role in cancer progression [11, 12]. Notably, FOXA2 expression is downregulated in nearly all lung cancer cell lines [13]. Moreover, previous studies have reported that FOXA2 promotes the transcription of downstream target genes [14]. Using data from the NCBI and JASPAR databases, we identified putative FOXA2 binding sites in the promoter region of ACSL4. However, the regulatory relationship between FOXA2 and ACSL4 in NSCLC remains largely uncharacterized.

Deubiquitinases (DUBs) have received increasing attention for their involvement in cancer‐related signaling pathways, owing to their diverse functional roles [15]. Ubiquitin‐specific protease 54 (USP54), a member of the ubiquitin‐specific protease family, has been implicated in the pathogenesis of various cancers, including prostate cancer, gastric cancer, and lung adenocarcinoma [16, 17, 18]. Preliminary analysis using the GEPIA database revealed decreased USP54 expression in NSCLC. Predictive data from Ubibrower 2.0 indicated that USP54 may act as a deubiquitinase for FOXA2 and that a protein–protein interaction exists between them. Nevertheless, the regulatory interplay between USP54 and FOXA2 in NSCLC has yet to be elucidated.

In our study, we conducted a series of experiments to test the hypothesis that USP54 enhances the stability of FOXA2 via deubiquitination, thereby upregulating ACSL4 expression and promoting ferroptosis in NSCLC cells. Our results demonstrate that USP54 exerts a tumor‐suppressive function in NSCLC by facilitating ferroptosis through FOXA2‐dependent transcriptional activation of ACSL4. This newly identified regulatory mechanism not only advances the current understanding of ferroptosis in lung cancer but also highlights a potential therapeutic target. Modulating the USP54–FOXA2–ACSL4 axis may enhance ferroptotic sensitivity in NSCLC, offering promising implications for improving treatment outcomes and patient prognosis.

## 2 Materials and Methods

### 2.1 Sample Collection

Before enrolling in the study, written informed consent was obtained from all participants. This research was carried out according to ethical principles and received approval from the Ethics Committee of the hospital (IRB: 2024 [SR] NO. 010). The research team implemented extensive measures to safeguard participants' rights, privacy, and confidentiality throughout the study. All the data were securely stored and analyzed anonymously, with each participant assigned a unique identification code to ensure confidentiality. Notably, none of the participants had received radiotherapy or preoperative chemotherapy. Detailed patient information, including the source, number of patients, and relevant clinical characteristics, is provided in Table S1. For this study, both NSCLC tissues and adjacent normal tissues were collected from 20 patients diagnosed with NSCLC. The tissue samples were meticulously dissected and immediately preserved in liquid nitrogen for future experimental analyses.

### 2.2 Public Database Analysis

USP54 expression data for lung squamous cell carcinoma (LUSC) were obtained from the GEPIA database, which combines data from the TCGA and Genotype‐Tissue Expression (GTEx) databases. Survival data were retrieved from the Kaplan–Meier plotter, where patients were divided into high and low USP54 expression groups on the basis of the median value. Kaplan–Meier survival curves were generated, and hazard ratios (HRs) with 95% confidence intervals (CIs) and log‐rank *p* values were calculated by the Kaplan–Meier plotter to assess the associations between USP54 expression levels and overall survival in LUSC patients.

### 2.3 Cell Culture

The human bronchial epithelial cell line (BEAS‐2B) and NSCLC cell lines (A549, SK‐MES‐1, H1975, PC19) were sourced from the American Type Culture Collection (ATCC). All the cell lines were maintained in RPMI 1640 medium (Gibco, Carlsbad, CA, USA) enriched with 10% fetal bovine serum (FBS, Gibco), glutamine, 100 U/mL penicillin, and 100 μg/mL streptomycin (Gibco). The cells were incubated in a controlled environment with 5% CO₂ at 37°C. For cell treatment, 2.5 μM erastin (Sigma–Aldrich, St. Louis, USA, cat. no. S7242) was added to the culture medium, and cell viability was evaluated after 24 h. To inhibit ferroptosis, the cells were treated with 10 μM ferrostatin‐1 (Fer‐1; Sigma–Aldrich, cat. no. SML0583) for 24 h.

### 2.4 Cell Transfection

To examine the impact of gene silencing and overexpression on non‐small cell lung cancer (NSCLC) cells, a series of transfection and infection experiments was conducted. NSCLC cells were initially plated in 6‐well plates and incubated for 24 h to allow for adhesion before transfection. Short hairpin RNAs (shRNAs) targeting USP54 (sh‐USP54), FOXA2 (sh‐FOXA2), and a nontargeting control (sh‐NC) were synthesized by Genesee Biotech and inserted into GV102 vectors (Genepharma, Shanghai, China). For gene overexpression studies, the full‐length cDNAs of USP54 and ACSL4 were amplified via PCR and cloned and inserted into the pcDNA3.1 vector (Promega, Madison, WI, USA), resulting in the generation of USP54 (oe‐USP54) and ACSL4 (oe‐ACSL4) overexpression plasmids. An empty pcDNA3.1 vector served as the negative control (oe‐NC). Transfections were performed using 20 μL of Lipofectamine 3000 (Invitrogen, CA, USA) per well, with a total of 5 × 10^6^ cells. After 6 h, the transfection medium was replaced with fresh culture medium, and the cells were incubated for an additional 48 h to ensure gene expression. These transfected cells were then utilized in various in vitro functional assays to assess their cellular behavior.

### 2.5 RNA Extraction and Quantitative Real‐Time Polymerase Chain Reaction (qPCR)

The cells were lysed, and total RNA was isolated using TRIzol reagent (Invitrogen) following the instructions provided by the manufacturer. Subsequently, 1 μg RNA was reverse transcribed into complementary DNA (cDNA) using the PrimeScript cDNA Synthesis Kit (Takara, Osaka, Japan). qPCR was conducted using the TaqMan Universal PCR Master Mix (Thermo Fisher Scientific, cat. no. 4305719) to amplify the target genes. The specific primers used for each reaction are detailed in Table 1. Relative RNA expression levels were determined using the 2^−ΔΔCt^ method, with GAPDH employed as the internal reference for normalization.

**TABLE 1:** Primers used for RT–qPCR.

Gene name	Forward sequence	Reverse sequence
USP54	GAGTTAGAGGCAGCGAAAGGGT	TCTTGCAGGGACCTCTCAAAGC
SLC7A11	TCCTGCTTTGGCTCCATGAACG	AGAGGAGTGTGCTTGCGGACAT
GPX4	ACAAGAACGGCTGCGTGGTGAA	GCCACACACTTGTGGAGCTAGA
FOXA2	GGAACACCACTACGCCTTCAAC	AGTGCATCACCTGTTCGTAGGC
ACSL4	GCTATCTCCTCAGACACACCGA	AGGTGCTCCAACTCTGCCAGTA
GAPDH	GTCTCCTCTGACTTCAACAGCG	ACCACCCTGTTGCTGTAGCCAA
ACSL4‐promoter	ACCCAGTGAATTACGTACCTGAC	AGTTCAGTACTTCAAACAACGAGT

### 2.6 Western Blot

Proteins were extracted from the cell samples by incubation in radioimmunoprecipitation assay (RIPA) buffer containing a protease inhibitor cocktail for 30 min at 4°C (Beyotime Inc., Haimen, Jiangsu, China). The protein concentration was measured by a bicinchoninic acid (BCA) assay kit from Thermo Fisher Scientific (cat. no. 10741395, Männedorf, Switzerland). Equal amounts of protein (30 μg per sample) were resolved by sodium dodecyl sulfate–polyacrylamide gel electrophoresis (SDS–PAGE) and subsequently transferred onto polyvinylidene fluoride (PVDF) membranes. The membranes were blocked, rinsed with PBS, and incubated with primary antibodies targeting solute carrier family 7 member 11 (SLC7A11, Thermo Fisher Scientific, cat. no. PA1‐16893, 1:1000), glutathione peroxidase 4 (GPX4, Thermo Fisher Scientific, cat. no. MA5‐32827, 1:10000), ACSL4 (Thermo Fisher Scientific, cat. no. PA5‐27137, 1:10000), FOXA2 (Thermo Fisher Scientific, cat. no. MA5‐15542, 1:1000), USP54 (Thermo Fisher Scientific, cat. no. PA5‐103905, 1:1000), and GAPDH (Thermo Fisher Scientific, cat. no. MA5‐15738, 1:10000). After washing with PBS, the membranes were incubated with secondary antibodies (1:2000, Invitrogen, cat. no. 31402). The protein bands were visualized using an enhanced chemiluminescence (ECL) detection system (Merck Millipore, Billerica, USA; cat. no. WBULS0100).

### 2.7 Immunohistochemistry (IHC) Staining

The tumor tissues were cut into 5 μm‐thick sections. The sections were deparaffinized and rehydrated, followed by antigen retrieval through heating in citrate buffer. To inhibit endogenous peroxidase activity, the tissues were treated with hydrogen peroxide. After being blocked with goat serum, the sections were incubated at 4°C for 12 h with primary antibodies against USP54 (Thermo Fisher Scientific, cat. no. PA5‐103905, 1:1000). Following PBS washes, the sections were incubated with secondary antibodies for 1 h at room temperature. Hematoxylin was applied as a counterstain, and the sections were mounted for analysis. The stained tissues were then examined and imaged using a DMi8 optical microscope (Leica, Wetzlar, Germany).

### 2.8 3‐(4,5‐Dimethylthiazol‐2‐yl)‐2,5‐Diphenyltetrazolium Bromide (MTT) Assay

Cells were seeded into 96‐well plates at a density of 5 × 10^3^ cells per well. After cells were incubated for 0, 12, 24, or 48 h, 0.5 mg/mL MTT solution (Sigma–Aldrich) was added to each well and incubated for approximately 4 h. The absorbance was measured at 570 nm using a Bio‐Rad microplate reader‐550 (Bio‐Rad, Shanghai, China) to assess cell viability.

### 2.9 Colony Formation Assay

Cells were trypsinized and seeded into 6‐well plates at a density of 500 cells per well. The cells were cultured for approximately 14 days to allow colony formation, and the media were changed every 3 days. Colonies were then fixed with 4% paraformaldehyde for 15 min and stained with 0.5% crystal violet for 20 min.

### 2.10 Detection of Malondialdehyde (MDA), Fe^2+^, and Reactive Oxygen Species (ROS)

Cells (2 × 10^6^) were washed with PBS and then trypsinized for further analysis. The levels of MDA, iron (Fe^2+^), and ROS in the samples were quantified using the following specific assay kits: the Lipid Peroxidation Assay Kit (Abcam, Cambridge, UK, cat. no. ab118970), the Iron Assay Kit (Abcam, cat. no. ab83366), and the ROS Assay Kit (Abcam, cat. no. ab186027). All the assays were performed according to the protocols provided by the respective manufacturers.

### 2.11 Detection of Protein Stability

To assess protein stability, A549 cells were transfected with sh‐NC or sh‐USP54. Following transfection, the cells were treated with cycloheximide (CHX; Aladdin, Shanghai, China) at a final concentration of 50 μg/mL for various durations (0, 2, 4, 6, and 8 h). At each time point, FOXA2 protein levels were quantified using a Western blot.

### 2.12 Co‐Immunoprecipitation (Co‐IP) Assay

A549 cells were first rinsed with ice‐cold PBS and then lysed with NP‐40 buffer supplemented with a protease inhibitor cocktail. The lysates were kept on ice for 15 min and subsequently centrifuged at 15,000 × *g* for 15 min to remove any insoluble material. The supernatants were then incubated with protein G‐Sepharose at 4°C for 15 min to eliminate nonspecific proteins. Following this preclearing step, the samples were incubated overnight at 4°C with anti‐USP54 antibody (Thermo Fisher Scientific, 1:50, cat. no. PA5‐103905), anti‐FOXA2 antibody (Thermo Fisher Scientific, 1:50, cat. no. MA5‐42678), or a control IgG antibody (Abcam, 1:1000, cat. no. ab18413). The next day, immune complexes were captured by adding protein G‐Sepharose beads and incubating for an additional 2 h at 4°C. The beads were then washed four times with lysis buffer, resuspended in 4 × loading buffer, and boiled for 5 min to denature the bound proteins. Finally, the proteins were measured by western blot.

### 2.13 Dual‐Luciferase Reporter Assay

The wild‐type ACSL4 (ACSL4‐wt) and its mutant variant (ACSL4‐mut) were cloned and inserted into the pGL3‐Basic vector (Promega) after PCR amplification. A549 cells were then transfected with either ACSL4‐wt or ACSL4‐mut, along with oe‐FOXA2 or vector, using Lipofectamine 3000 (Invitrogen) for transfection. Following 48 h of incubation, luciferase activity was measured using the Dual‐Luciferase Reporter Assay Kit (Promega).

### 2.14 Chromatin Immunoprecipitation (ChIP) Assay

A549 cells were harvested and lysed in RIP lysis buffer (Merck Millipore). The cells were fixed with 1% formaldehyde for 10 min at room temperature to cross‐link protein–DNA complexes, and the cross‐linking was subsequently quenched with 125 mM glycine. After lysis, the nuclei were isolated, and chromatin was sheared via sonication to generate DNA fragments ranging from 200 to 500 bp in length. The sheared chromatin was precleared with protein A/G magnetic beads and then incubated overnight at 4°C with an anti‐FOXA2 antibody (Thermo Fisher Scientific, cat. no. MA5‐42678, 1:50) or an anti‐IgG antibody (Abcam, cat. no. ab205718, 1:1000). Immunocomplexes were captured with magnetic beads, followed by sequential washes with different buffers. The chromatin–protein complexes were eluted via elution buffer for 30 min, and the cross‐links were reversed overnight at 65°C in the presence of 200 mM NaCl. The resulting DNA was purified using a PCR purification kit (Qiagen, Hilden, Germany) and subsequently analyzed by qPCR. The ChIP primer sequence is shown in Table 1.

### 2.15 Statistical Analysis

The results are reported as the mean ± standard deviation (SD) of at least three independent experiments. The data distribution was assessed by Shapiro–Wilk. The data were analyzed using SPSS software, version 20.0 (IBM, Armonk, NY, USA). Statistical differences between two groups were analyzed using a paired or unpaired Student's *t*‐test, whereas comparisons involving more than two groups were performed using one‐way analysis of variance (ANOVA) followed by Tukey's post hoc test. Pearson correlation analysis was used to examine the correlation between the expression of USP54 and FOXA2, SLC7A11, GPX4, or ACSL4. A *p* value of less than 0.05 was considered statistically significant.

## 3 Results

### 3.1 USP54 Expression Is Downregulated in NSCLC

**FIGURE 1:**
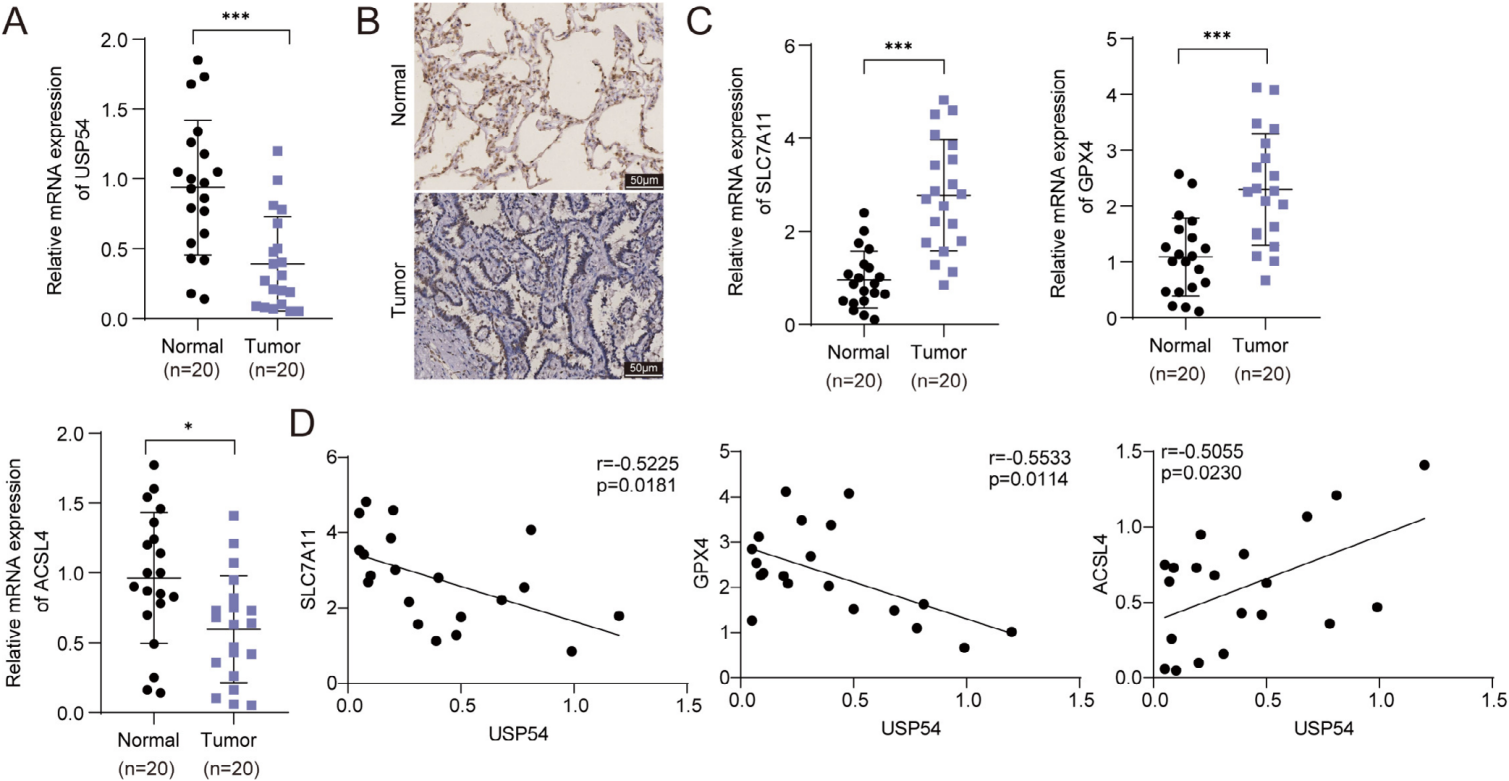
USP54 expression is downregulated in NSCLC. (A) qPCR analysis was performed to assess USP54 expression levels in NSCLC samples and adjacent normal (*n* = 20). Statistical significance was determined using the paired *t*‐test. (B) Immunohistochemistry (IHC) was used to detected the expression of USP54 in representative NSCLC tumor and adjacent normal tissues. Scale bars: 50 μm. (C) qPCR analysis was used to measure the expression of ferroptosis‐related genes, including SLC7A11, GPX4, and ACSL4, in NSCLC tumor tissues and normal tissues (*n* = 20). Statistical significance was determined using the paired *t*‐test. (D) Correlation analysis was conducted to evaluate the relationships between USP54 and ferroptosis‐related genes, including SLC7A11, GPX4, and ACSL4. The data are presented as the means ± SD. *p < 0.05, **p < 0.01, ***p < 0.001.

To investigate the expression of USP54 in NSCLC, we first analyzed USP54 expression from public datasets. It revealed that USP54 expression was significantly decreased in lung cancer tissues compared with normal lung tissues (Figure S1A), and higher USP54 expression was associated with improved patient prognosis (Figure S1B). These findings were further validated by qPCR and IHC, both of which confirmed reduced USP54 expression in NSCLC tissues (Figure 1A,B). Ferroptosis, an iron‐dependent form of cell death, is regulated by key molecules such as GPX4 and SLC7A11 [19]. Therefore, we examined their expression in NSCLC tissues. The results showed that SLC7A11 and GPX4 levels were elevated, whereas ACSL4 was decreased in NSCLC tumor tissues compared to adjacent normal tissues (Figure 1C). Correlation analysis further indicated that USP54 expression was negatively associated with SLC7A11 and GPX4 but positively correlated with ACSL4 (Figure 1D). In summary, USP54 expression is markedly reduced in NSCLC.

### 3.2 Overexpression of USP54 Suppresses NSCLC Cell Proliferation and Migration While Promoting Ferroptosis

**FIGURE 2:**
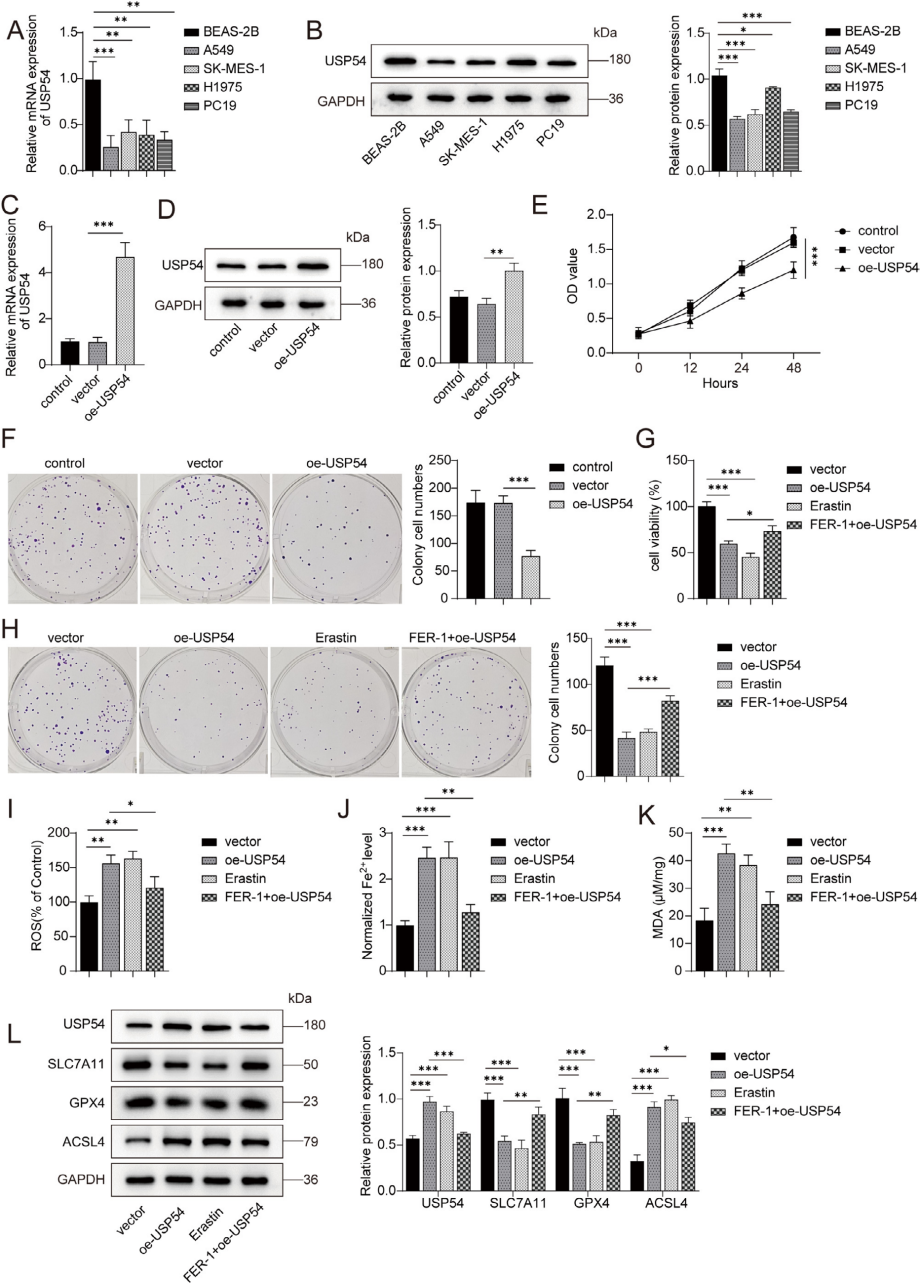
Overexpression of USP54 suppresses NSCLC cell proliferation and migration while promoting ferroptosis. (A, B) qPCR and Western blot analysis were performed to measure USP54 expression in the NSCLC cell lines A549, SK‐MES‐1, H1975, and PC19 and the human bronchial epithelial cell line BEAS‐2B (*n* = 3). Statistical significance was determined using one‐way ANOVA followed by Tukey's post hoc test. (C, D) qPCR and Western blotting were used to detect USP54 expression in A549 cells transfected with vector or oe‐USP54 (*n* = 3). Statistical significance was determined using one‐way ANOVA followed by Tukey's post hoc test. (E) Cell viability was assessed using the MTT assay (*n* = 3). Statistical significance was determined using two‐way ANOVA followed by Tukey's post hoc test. (F) Colony formation assays were conducted to evaluate the proliferation of A549 cells transfected with vector or oe‐USP54 (*n* = 3). Statistical significance was determined using one‐way ANOVA followed by Tukey's post hoc test. A549 cells were transfected with or without oe‐USP54 and treated with or without erastin or FER‐1. (G) Cell viability was measured using the MTT assay (*n* = 3). Statistical significance was determined using one‐way ANOVA followed by Tukey's post hoc test. (H) Colony formation assays were conducted to evaluate cell proliferation (*n* = 3). Statistical significance was determined using one‐way ANOVA followed by Tukey's post hoc test. (I–K) ROS, Fe^2+^, and MDA levels were measured using assay kits to evaluate ferroptosis (*n* = 3). Statistical significance was determined using one‐way ANOVA followed by Tukey's post hoc test. (L) Western blot was performed to examine the expression of USP54 and ferroptosis‐related proteins, including SLC7A11, GPX4, and ACSL4 (*n* = 3). Statistical significance was determined using one‐way ANOVA followed by Tukey's post hoc test. The data are presented as the mean ± SD. *p < 0.05, **p < 0.01, ***p < 0.001.

To further explore the role of USP54 in NSCLC, we examined its expression in several NSCLC cell lines, including A549, SK‐MES‐1, H1975, and PC19. Compared with the human bronchial epithelial cell line BEAS‐2B, USP54 mRNA and protein expression were significantly reduced in NSCLC cell lines (Figure 2A,B). USP54 was then overexpressed in A549 and SK‐MES‐1 cells, with overexpression efficiency confirmed by qPCR and/or Western blot (Figures 2C,D and S2A). Overexpression of USP54 markedly suppressed cell viability and proliferation (Figures 2E,F and S2B,C). Furthermore, USP54 overexpression or erastin (a ferroptosis inducer) treatment reduced cell growth, while Fer‐1 (a ferroptosis inhibitor) promoted cell proliferation in lung cancer cells by oe‐USP54 treated (Figures 2G,H and S2D,E). USP54 overexpression and erastin treatment also significantly increased lipid ROS, Fe^2+^, and MDA levels, all of which were reversed by Fer‐1 treatment (Figures 2I–K and S2F–H). Western blot analysis showed that USP54 overexpression or erastin treatment reduced the expression of SLC7A11 and GPX4, while upregulating ACSL4 and USP54 levels (Figures 2L and S2I). These changes were reversed upon Fer‐1 treatment in oe‐USP54 treated lung cancer cells (Figures 2L and S2I). Taken together, USP54 overexpression inhibits NSCLC cell proliferation and migration and promotes ferroptosis.

### 3.3 USP54 Mediates the Deubiquitination and Regulation of FOXA2 Expression

**FIGURE 3:**
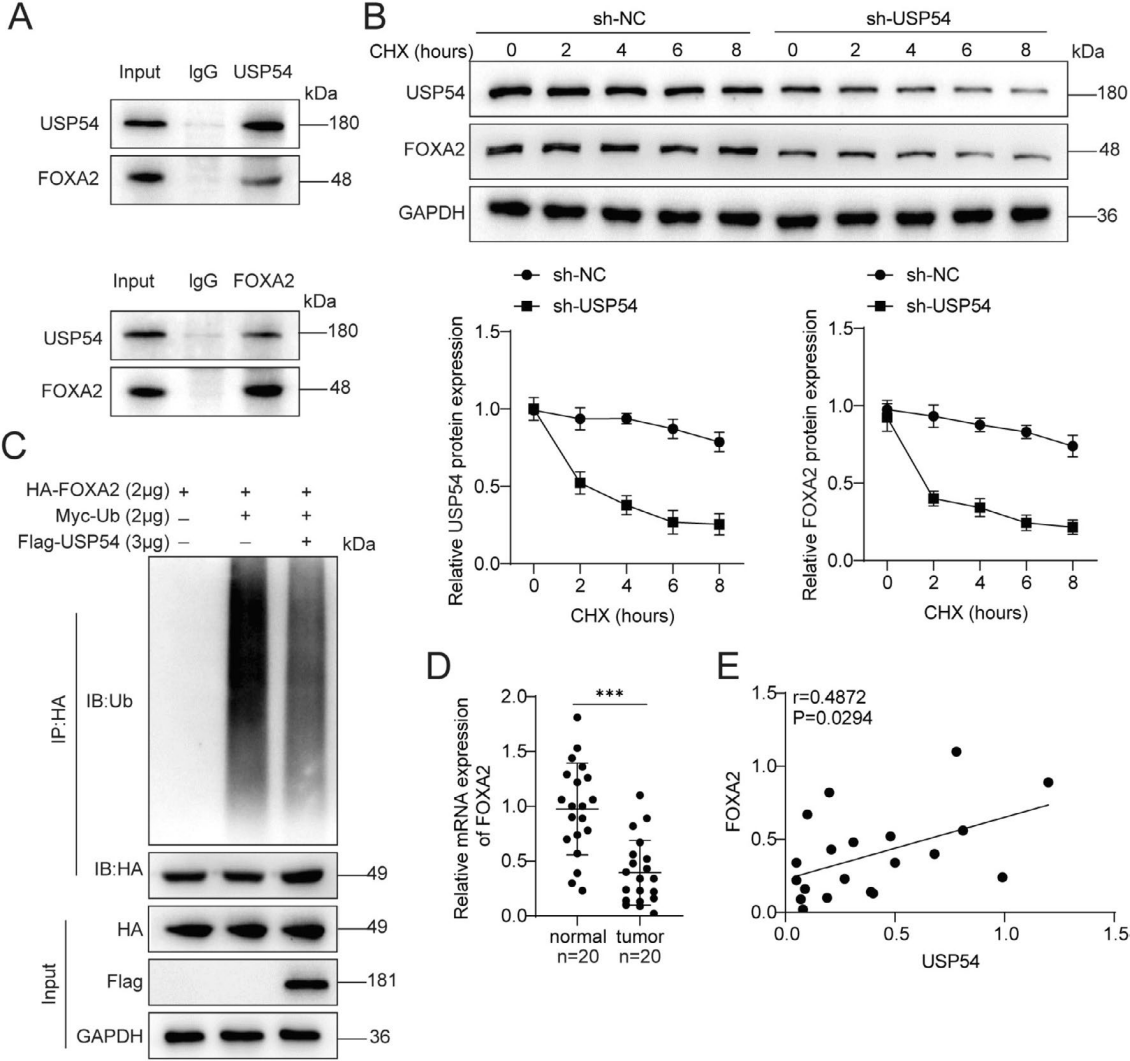
USP54 mediates the deubiquitination and regulation of FOXA2 expression. (A) Coimmunoprecipitation (co‐IP) assays were conducted to confirm the interaction between USP54 and FOXA2 in A549 cells (*n* = 3). (B) Western blot was performed to evaluate FOXA2 and USP54 expression following USP54 knockdown in CHX‐treated A549 cells (*n* = 3). Statistical significance was determined using one‐way ANOVA followed by Tukey's post hoc test. (C) Ubiquitination assays were used to assess the ubiquitination of FOXA2 (*n* = 3). (D) FOXA2 expression was assessed in NSCLC tissues and normal tissues (*n* = 20). Statistical significance was determined using the paired *t*‐test. (E) Pearson correlation analysis was performed to evaluate the relationship between FOXA2 and USP54. The data are presented as the mean ± SD. *p < 0.05, **p < 0.01, ***p < 0.001.

USP54 is a member of the USP family and is known to function as a deubiquitinase that regulates the stability of various substrate proteins [16]. Bioinformatic predictions from the Ubibrowser 2.0 database identified FOXA2 as one of the potential ubiquitinated substrates of USP54. To investigate the downstream mechanism of USP54 in NSCLC, we performed Co‐IP assays, which confirmed that USP54 physically interacted with FOXA2 in A549 cells (Figure 3A). Furthermore, knockdown of USP54 significantly reduced the stability of FOXA2, as demonstrated by accelerated degradation of FOXA2 in CHX‐treated cells in a time‐dependent manner (Figure 3B). Additionally, USP54 decreased the ubiquitination levels of FOXA2 (Figure 3C), supporting its role as a deubiquitinase. FOXA2 expression was also found to be downregulated in NSCLC tissues compared with adjacent normal tissues (Figure 3D), and correlation analysis revealed a positive association between USP54 and FOXA2 expression (Figure 3E). Collectively, USP54 stabilizes FOXA2 expression by mediating its deubiquitination, thereby playing a critical regulatory role in NSCLC.

### 3.4 USP54 Enhances Ferroptosis in NSCLC by Suppressing the Degradation of FOXA2

**FIGURE 4:**
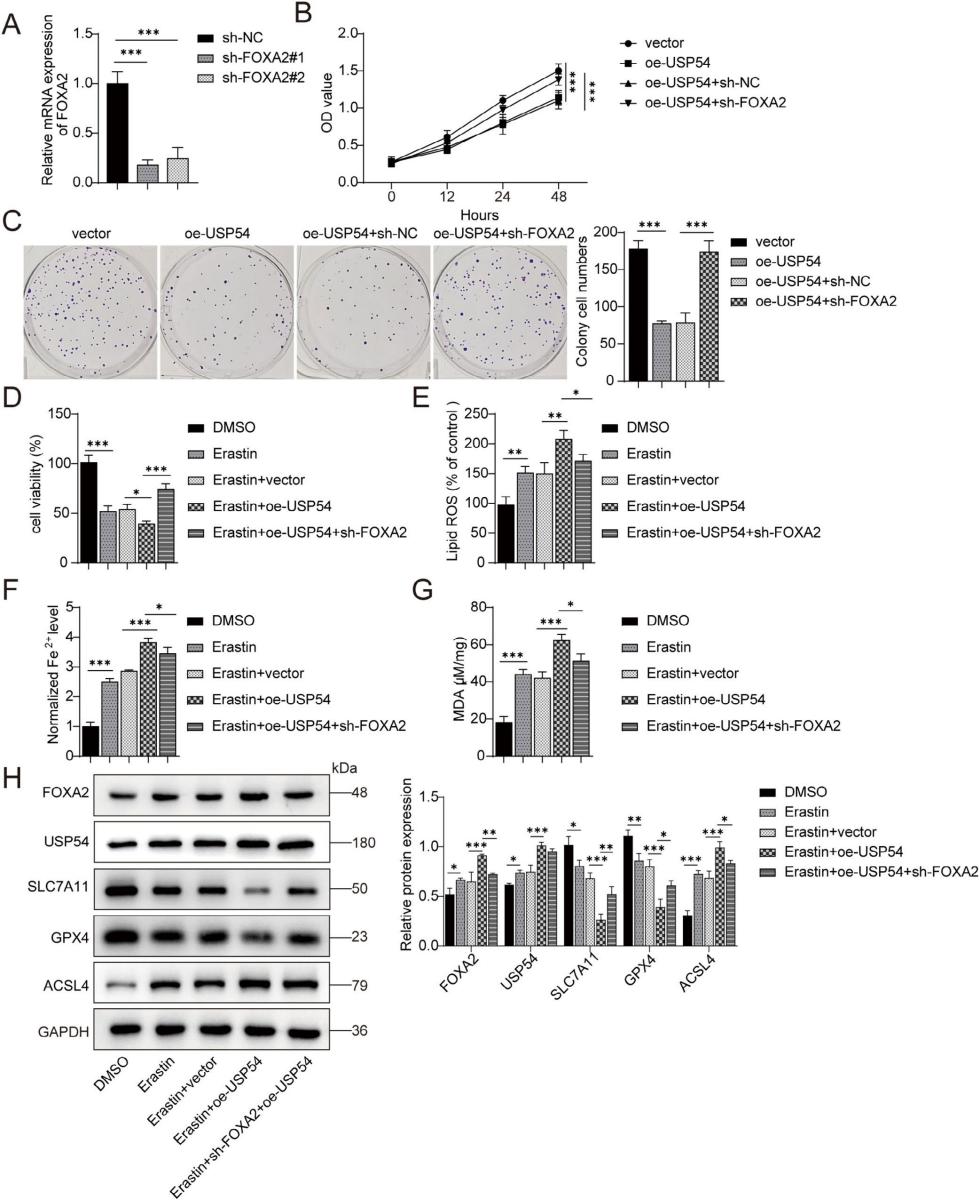
USP54 enhances ferroptosis in NSCLC by suppressing the ubiquitination of FOXA2. (A) qPCR analysis was performed to detect the expression of FOXA2 in A549 cells transfected with sh‐NC or sh‐FOXA2 (*n* = 3). Statistical significance was determined using Student's *t*‐test. (B) MTT and (C) colony formation assays were conducted to assess the viability and proliferation of A549 cells transfected with vector, oe‐USP54, oe‐USP54 + sh‐NC, or oe‐USP54 + sh‐FOXA2 (*n* = 3). Statistical significance was determined using one‐way ANOVA followed by Tukey's post hoc test. A549 cells were treated with DMSO, erastin, erastin + vector, erastin + oe‐USP54, or erastin + oe‐USP54 + sh‐FOXA2. (D) Cell viability was tested by MTT assay. (E) The ROS levels, (F) Fe^2+^ levels, and (G) MDA levels were measured using biochemical assay kits (*n* = 3). Statistical significance was determined using one‐way ANOVA followed by Tukey's post hoc test. (H) Western blot was performed to assess the expression of USP54, FOXA2, SLC7A11, GPX4, and ACSL4 in A549 cells (*n* = 3). Statistical significance was determined using one‐way ANOVA followed by Tukey's post hoc test. The data are presented as the mean ± SD. *p < 0.05, **p < 0.01, ***p < 0.001.

To further elucidate the role of FOXA2 in USP54‐mediated regulation of NSCLC progression, we silenced FOXA2 and/or overexpressed USP54 in A549 cells. qPCR confirmed efficient FOXA2 knockdown (Figure 4A). Overexpression of USP54 significantly reduced cell viability and proliferation; however, these inhibitory effects were reversed upon FOXA2 silencing (Figure 4B,C). Additionally, USP54 overexpression enhanced the inhibitory effects of erastin on cell viability, but this increase was mitigated by FOXA2 knockdown (Figure 4D). In the presence of erastin, USP54 overexpression markedly increased lipid ROS, Fe^2+^, and MDA levels, which were attenuated by FOXA2 knockdown (Figure 4E–G). Western blot analysis further revealed that USP54 overexpression amplified erastin‐induced downregulation of SLC7A11 and GPX4 and upregulation of ACSL4, whereas FOXA2 knockdown reversed these protein expression changes (Figure 4H). In addition, USP54 overexpression increased USP54 and FOXA2 expression in erastin‐induced cells, but sh‐FOXA2 transfection reduced FOXA2 expression (Figure 4H). Together, USP54 promotes ferroptosis and inhibits cell proliferation by stabilizing FOXA2.

### 3.5 FOXA2 Promotes ACSL4 Transcription

**FIGURE 5:**
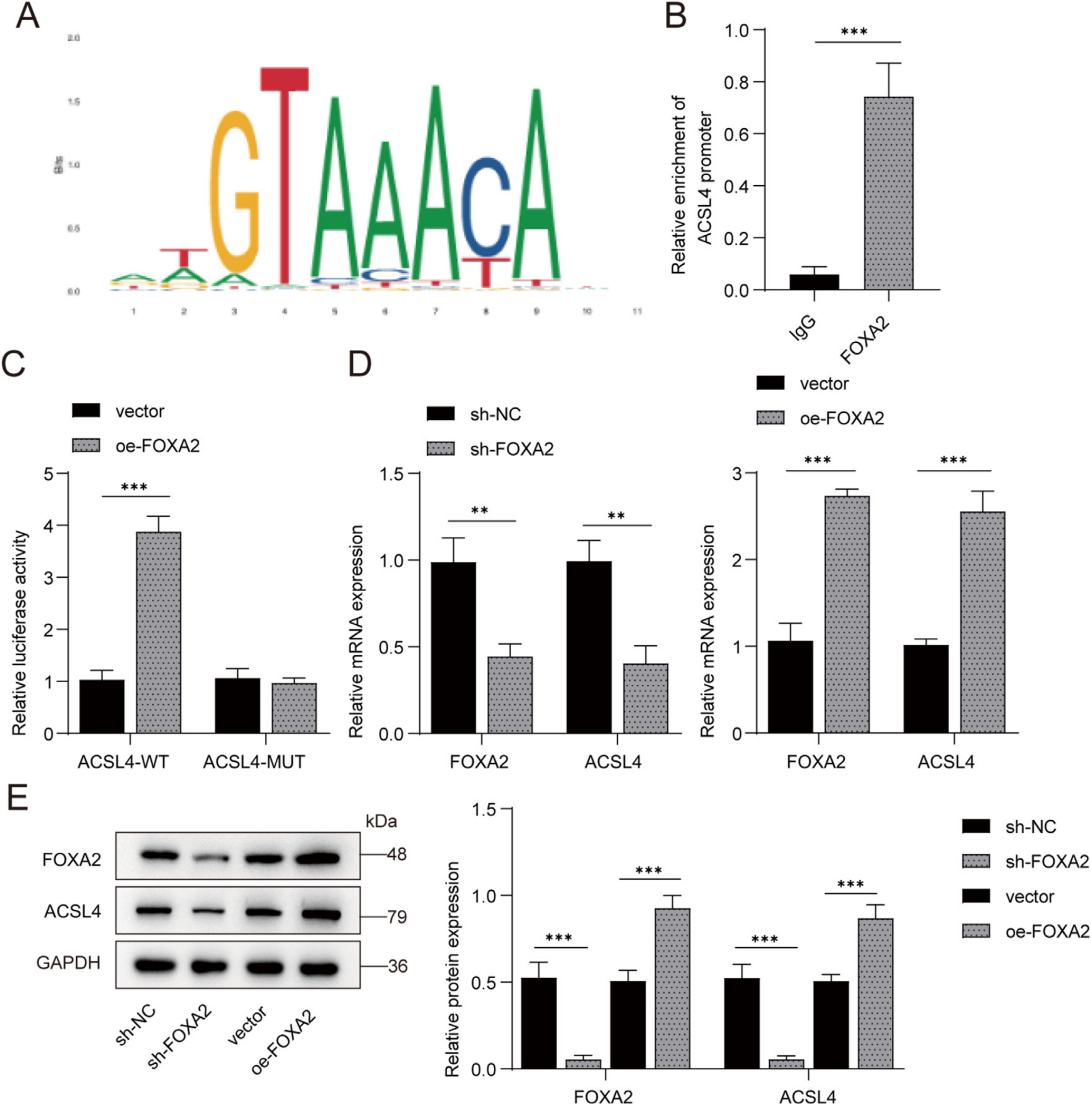
FOXA2 promotes ACSL4 transcription. (A) The potential binding sites in the ACSL4 promoter region. (B) ChIP was performed to test the interaction between FOXA2 and ACSL4 (*n* = 3). Statistical significance were analyzed using Student's *t*‐test. (C) Dual‐luciferase reporter assays were conducted to evaluate luciferase activity (*n* = 3). Statistical significance was performed using Student's *t*‐test. (D) qPCR was performed to measure the expression levels of FOXA2 and ACSL4 in A549 cells (*n* = 3). Statistical significance were analyzed using Student's *t*‐test. (E) Western blot was used to detect the protein levels of FOXA2 and ACSL4 in A549 cells (*n* = 3). Statistical significance were analyzed using Student's *t*‐test. The data are presented as the mean ± SD. **p < 0.01, ***p < 0.001.

FOXA2 is a member of the FOX transcription factor family and plays a pivotal role in regulating gene expression involved in tumor development [20, 21]. Bioinformatics analysis revealed a putative FOXA2 binding site in the promoter region of ACSL4 (Figure 5A). This prediction was experimentally validated by ChIP assays, which showed increased enrichment of FOXA2 at the ACSL4 promoter when an anti‐FOXA2 antibody was used (Figure 5B). Additionally, FOXA2 overexpression significantly enhanced luciferase activity in cells transfected with the wild‐type ACSL4 promoter construct, while no significant change was observed in the mutant construct lacking the FOXA2 binding site (Figure 5C). FOXA2 knockdown led to a reduction in both FOXA2 and ACSL4 expression levels, and FOXA2 overexpression increased FOXA2 and ACSL4 expression (Figure 5D,E). Overall, FOXA2 positively regulates ACSL4 transcription in NSCLC cells by directly binding to its promoter region.

### 3.6 USP54 Enhances Ferroptosis in NSCLC by Regulating FOXA2/ACSL4 Axis

**FIGURE 6:**
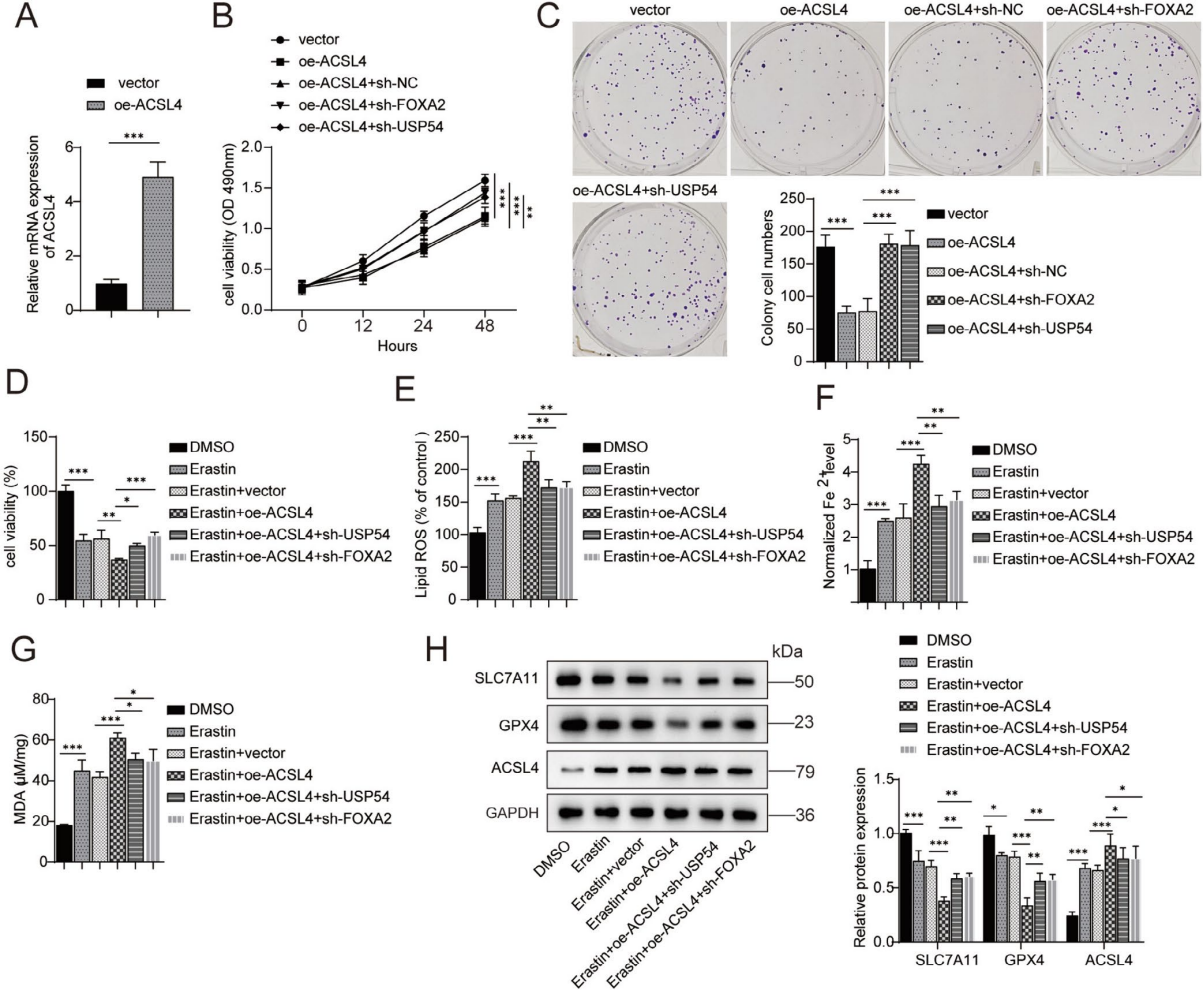
USP54 promotes ferroptosis in NSCLC by regulating the FOXA2/ACSL4 axis. (A) qPCR was performed to detect the expression of ACSL4 in A549 cells transfected with vector or oe‐ACSL4 (*n* = 3). Statistical significance were analyzed using Student's *t*‐test. (B, C) A549 cells were transfected with oe‐ACSL4, sh‐FOXA2 and sh‐USP54, and the groups as follows: Vector, oe‐ACSL4, oe‐ACSL4 + sh‐NC, oe‐ACSL4 + sh‐FOXA2, or oe‐ACSL4 + sh‐USP54. MTT and colony formation assays were conducted to assess cell viability and proliferation (*n* = 3). Statistical significance was determined using one‐way ANOVA followed by Tukey's post hoc test. (D–G) Cells are grouped into DMSO, erastin, erastin + vector, erastin + oe‐ACSL4, erastin + oe‐ACSL4 + sh‐USP54, or erastin + oe‐ACSL4 + sh‐FOXA2. (D) Cell viability was tested by MTT assay (*n* = 3). Statistical significance was determined using one‐way ANOVA followed by Tukey's post hoc test. ROS levels (E), Fe^2+^ levels (F), and MDA levels (G) were measured using respective biochemical assay kits (*n* = 3). Statistical significance was determined using one‐way ANOVA followed by Tukey's post hoc test. (H) Western blot was performed to examine the expression of SLC7A11, GPX4, and ACSL4 (*n* = 3). Statistical significance was determined using one‐way ANOVA followed by Tukey's post hoc test. The data are presented as the means ± SDs. *p < 0.05, **p < 0.01, ***p < 0.001.

The further investigate the regulatory role of ACSL4 in USP54‐ and FOXA2‐mediated NSCLC progression, we overexpressed ACSL4 while silencing FOXA2 or USP54 in A549 cells. qPCR confirmed the successful overexpression of ACSL4 (Figure 6A). ACSL4 overexpression significantly reduced cell viability and proliferation; however, these inhibitory effects were reversed by the knockdown of either FOXA2 or USP54 (Figure 6B,C). Moreover, ACSL4 overexpression enhanced the erastin‐induced suppression of cell viability, which was also reversed upon FOXA2 or USP54 knockdown (Figure 6D). In the presence of erastin, ACSL4 overexpression increased lipid ROS, Fe^2+^, and MDA levels, all of which were mitigated by FOXA2 or USP54 knockdown (Figure 6E–G). Western blot analysis further demonstrated that ACSL4 overexpression augmented erastin‐induced downregulation of SLC7A11 and GPX4, along with upregulation of ACSL4 itself (Figure 6H). These regulatory effects were abolished by FOXA2 or USP54 knockdown (Figure 6H). Taken together, USP54 enhances ferroptosis and suppresses NSCLC proliferation via the FOXA2/ACSL4 axis.

## 4 Discussion

NSCLC is the most common prevalent form of lung cancer and is associated with poor prognosis despite advancements in surgical techniques and chemotherapeutic strategies [22]. The 5‐year survival rate remains below 20%, and patients frequently experience severe chemotherapy‐related toxicity and a high risk of disease recurrence [22]. Therefore, the identification of novel therapeutic targets for NSCLC is urgently needed. In this study, we provided new insights into the role of USP54 in regulating ferroptosis in NSCLC. We demonstrated that USP54 expression was downregulated in NSCLC and its overexpression suppressed NSCLC cell proliferation and induced ferroptosis. Furthermore, we showed that USP54 promoted the deubiquitination and stabilization of FOXA2, thereby enhancing the transcription of ACSL4, a key mediator of ferroptosis in NSCLC cells. These findings might provide a theoretical basis for the development of therapies targeting USP54 or ACSL4 for the treatment of NSCLC.

USP54 is a deubiquitinase that plays a critical role in maintaining protein stability by removing ubiquitin chains from substrate proteins, thereby preventing their proteasomal degradation [23]. In cancer, USP54 has been shown to influence tumor progression and metastasis, suggesting its potential as a therapeutic target [23]. For example, Chen et al. reported that USP54 expression was decreased in lung adenocarcinoma, and its overexpression inhibited aerobic glycolysis [18]. Ferroptosis, an iron‐dependent form of regulated cell death characterized by the accumulation of lipid peroxides, has emerged as a key mechanism of tumor suppression [19]. Central regulators of ferroptosis include GPX4 and SLC7A11: GPX4 mitigates lipid peroxidation by reducing lipid hydroperoxides, while SLC7A11 supports cystine uptake for glutathione synthesis, which is essential for maintaining GPX4 activity and redox balance [19]. Dysregulation of these ferroptosis‐related pathways has been implicated in cancer development and therapeutic resistance. In line with these findings, our study revealed that USP54 was downregulated in NSCLC. Functionally, USP54 overexpression significantly suppressed cell viability and proliferation while promoting ferroptosis in NSCLC, highlighting its potential tumor‐suppressive role through modulation of ferroptotic pathways.

Previous research has highlighted the role of USP54 in cancer, largely attributed to the capacity of deubiquitinases to regulate diverse protein substrates across various cell types [23]. In this study, we found that USP54 inhibited the ubiquitination of FOXA2, thereby preventing its proteasomal degradation and enhancing its protein stability. FOXA2 has been widely recognized as a key regulator in tumorigenesis and cancer progression. In lung cancer, FOXA2 is consistently reported to be downregulated in both cell lines and NSCLC tissues [13]. It has also been shown to cooperate with mutant Kirsten rat sarcoma viral oncogene homolog (KRAS) to promote the development of invasive mucinous adenocarcinoma of the lung [24]. Additionally, FOXA2 functions as a metastasis suppressor in lung cancer by inhibiting epithelial‐to‐mesenchymal transition [25]. Beyond lung cancer, FOXA2 has been implicated in the regulation of ferroptosis in other malignancies. In colorectal cancer, FOXA2 suppression by tripartite motif‐containing 36 induced ferroptosis via the NRF2/GPX4 signaling pathway [26]. In endometrial cancer, FOXA2 negatively regulated ACAT2, thereby promoting ferroptosis and inhibiting aggressive tumor phenotypes [27]. In the present study, we are the first to demonstrate that USP54 regulated FOXA2‐mediated ferroptosis in NSCLC. These findings provide novel insights into the tumor‐suppressive functions of USP54 and FOXA2 and underscore their potential as therapeutic targets in lung cancer.

ACSL4 plays a critical role in the regulation of ferroptosis and has been implicated in multiple cancer types [6]. In lung adenocarcinoma, ACSL4 expression is decreased [28]. Silencing ACSL4 enhanced the survival and invasiveness of lung cancer cells, whereas its overexpression promoted ferroptosis [28]. Additionally, ACSL4 has been reported to positively correlate with erastin‐induced ferroptosis in NSCLC cells [29]. Upregulation of ACSL4 leads to the accumulation of lipid peroxidation products and ROS, thereby sensitizing lung cancer cells to ferroptotic cell death [30]. As a result, ACSL4 has emerged as a promising therapeutic target for inducing ferroptosis in lung cancer. Consistent with these findings, our study demonstrated that FOXA2 transcriptionally regulated ACSL4 expression, thereby promoting ferroptosis and inhibiting NSCLC progression.

In conclusion, our study demonstrates that USP54 induces ferroptosis and inhibits NSCLC progression by stabilizing FOXA2, which subsequently promotes the transcription of ACSL4. This work uncovers a novel USP54–FOXA2–ACSL4 regulatory axis involved in ferroptosis and NSCLC pathogenesis. However, several limitations should be acknowledged. First, most of our experiments were performed in vitro, and further in vivo studies are required to validate these findings in a physiological context. Second, the potential crosstalk between ferroptosis and other forms of regulated cell death, which may influence the identified mechanisms, remains to be fully explored. Despite these limitations, our findings provide important insights into the molecular regulation of ferroptosis in lung cancer and highlight the USP54–FOXA2–ACSL4 axis as a promising therapeutic target for NSCLC treatment.

## Ethics Statement

This study was approved by the Ethics Committee of Changzhou Cancer Hospital (2024 [SR] NO. 010). The informed consent was obtained from study participants.

## Conflicts of Interest

The authors declare no conflicts of interest.

## Supporting information


**Figure S1:** (A, B) USP54 expression in lung squamous cell carcinoma (LUSC) tissues was analyzed using the GEPIA database, which integrates data from TCGA and the Genotype‐Tissue Expression (GTEx) project. The expression levels of USP54 were compared between tumor tissues and normal tissues, and statistical significance was determined using the default settings of GEPIA.

**Figure S2:** Overexpression of USP54 suppresses LUSC cell proliferation and migration while promoting ferroptosis. (A) qPCR was used to detect USP54 expression in SK‐MES‐1 cells transfected with vector or oe‐USP54 (*n* = 3). Statistical significance was determined using one‐way ANOVA followed by Tukey's post hoc test. (B) Cell viability in SK‐MES‐1 cells was assessed using the MTT assay (*n* = 3). Statistical significance was determined using one‐way ANOVA followed by Tukey's post hoc test. (C) Colony formation assays were conducted to evaluate the proliferation of SK‐MES‐1 cells (*n* = 3). Statistical significance was determined using one‐way ANOVA followed by Tukey's post hoc test. SK‐MES‐1 cells were transfected with or without oe‐USP54 and treated with or without erastin or FER‐1. (D, E) cell viability and proliferation were measured using the MTT and colony formation assay (*n* = 3). Statistical significance was determined using one‐way ANOVA followed by Tukey's post hoc test. (F–H) The ROS level, Fe^2+^ level, and MDA were measured using kits (*n* = 3). Statistical significance was determined using one‐way ANOVA followed by Tukey's post hoc test. (I) Western blot was performed to examine the expression of USP54, SLC7A11, GPX4, and ACSL4 (*n* = 3). Statistical significance was determined using one‐way ANOVA followed by Tukey's post hoc test. The data are presented as the means ± SDs. *p < 0.05, **p < 0.01, ***p < 0.001.

**Table S1:** Correlations between USP54 expression and clinicopathological variables.

## Data Availability

All data generated or analysed during this study are included in this article. The datasets used and/or analysed during the current study are available from the corresponding author on reasonable request.
