# Supplementary figures and images for: USP54 Promotes Ferroptosis in Non‐Small Cell Lung Cancer by Mediating FOXA2 Deubiquitination and Enhancing ACSL4 Transcription
Source: Kaohsiung J Med Sci. 2025 Nov 20;42(6):e70139. doi: 10.1002/kjm2.70139 (PMC13248803; doi:10.1002/kjm2.70139)

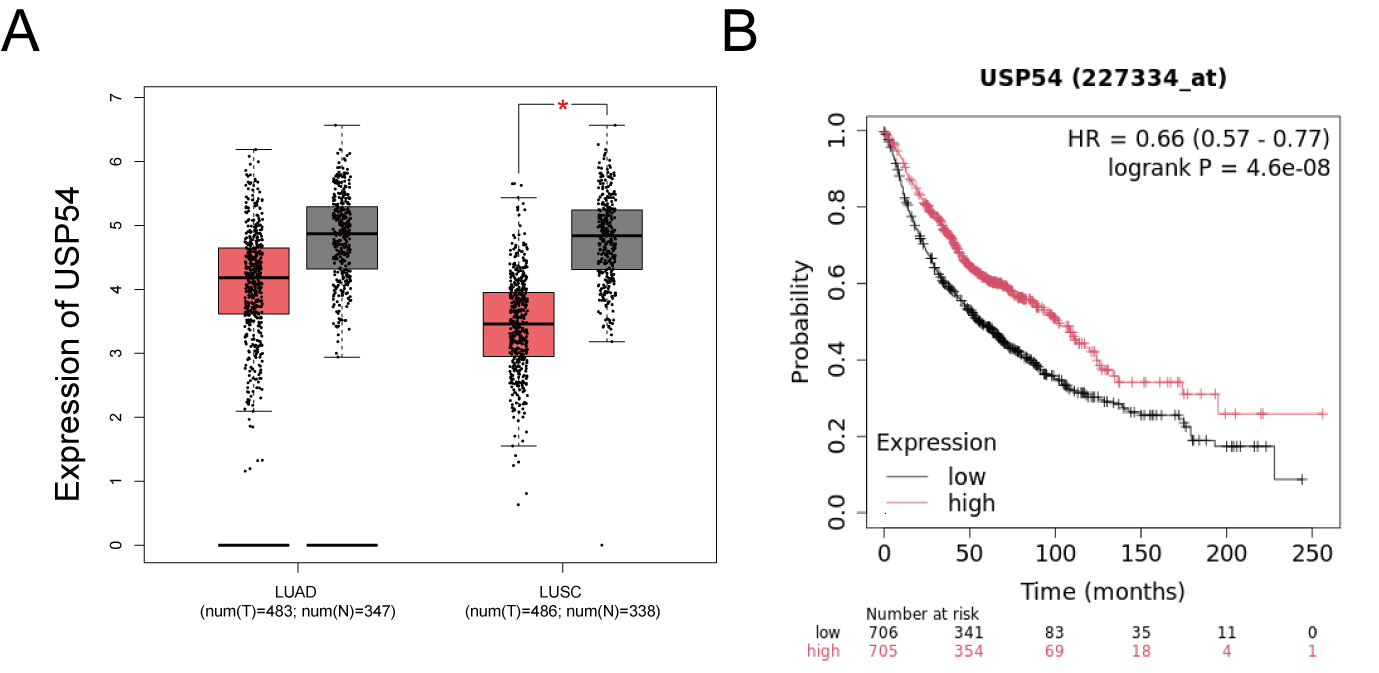

Supplement: Supplementary file 1 — Figure S1: (A, B) USP54 expression in lung squamous cell carcinoma (LUSC) tissues was analyzed using the GEPIA database, which integrates data from TCGA and the Genotype‐Tissue Expression (GTEx) project. The expression levels of USP54 were compared between tumor tissues and normal tissues, and statistical significance was determined using the default settings of GEPIA. [file KJM2-42-e70139-s001.tif]

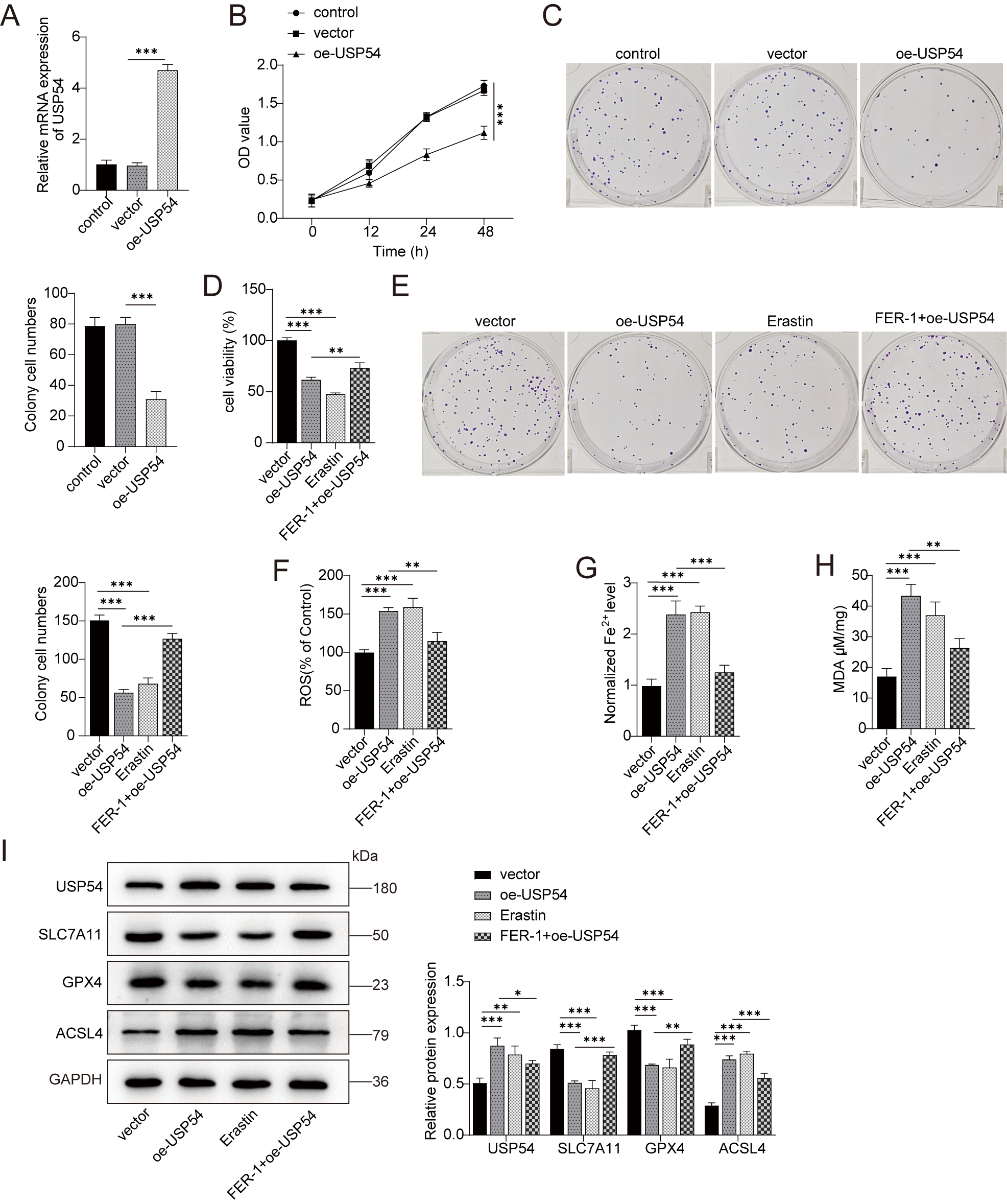

Supplement: Supplementary file 2 — Figure S2: Overexpression of USP54 suppresses LUSC cell proliferation and migration while promoting ferroptosis. (A) qPCR was used to detect USP54 expression in SK‐MES‐1 cells transfected with vector or oe‐USP54 (n = 3). Statistical significance was determined using one‐way ANOVA followed by Tukey's post hoc test. (B) Cell viability in SK‐MES‐1 cells was assessed using the MTT assay (n = 3). Statistical significance was determined using one‐way ANOVA followed by Tukey's post hoc test. (C) Colony formation assays were conducted to evaluate the proliferation of SK‐MES‐1 cells (n = 3). Statistical significance was determined using one‐way ANOVA followed by Tukey's post hoc test. SK‐MES‐1 cells were transfected with or without oe‐USP54 and treated with or without erastin or FER‐1. (D, E) cell viability and proliferation were measured using the MTT and colony formation assay (n = 3). Statistical significance was determined using one‐way ANOVA followed by Tukey's post hoc test. (F–H) The ROS level, Fe2+ level, and MDA were measured using kits (n = 3). Statistical significance was determined using one‐way ANOVA followed by Tukey's post hoc test. (I) Western blot was performed to examine the expression of USP54, SLC7A11, GPX4, and ACSL4 (n = 3). Statistical significance was determined using one‐way ANOVA followed by Tukey's post hoc test. The data are presented as the means ± SDs. *p < 0.05, **p < 0.01, ***p < 0.001. [file KJM2-42-e70139-s003.tif]
