# Supplementary material for: USP54 Promotes Ferroptosis in Non‐Small Cell Lung Cancer by Mediating FOXA2 Deubiquitination and Enhancing ACSL4 Transcription
Source: Kaohsiung J Med Sci. 2025 Nov 20;42(6):e70139. doi: 10.1002/kjm2.70139 (PMC13248803; doi:10.1002/kjm2.70139)
Supplement: Supplementary file 3 — Table S1: Correlations between USP54 expression and clinicopathological variables. [file KJM2-42-e70139-s002.docx]

**Table S1.** Correlations between USP54 expression and clinicopathological variables.

| **Variables** | **n** | **USP54 lower** | **USP54 higher** | **P** |
| --- | --- | --- | --- | --- |
| Age(years) |  |  |  | 0.311 |
| <60 | 11 | 6 | 5 |  |
| ≥60 | 9 | 3 | 6 |  |
| Gender |  |  |  | 0.608 |
| Male | 13 | 5 | 8 |  |
| Female | 7 | 3 | 4 |  |
| Smoking |  |  |  | 0.142 |
| No | 9 | 6 | 3 |  |
| Yes | 11 | 4 | 7 |  |
| Tumor size, cm |  |  |  | 0.078 |
| ≤3 | 14 | 11 | 3 |  |
| >3 | 6 | 2 | 4 |  |
| Distant metastasis |  |  |  | 0.562 |
| Yes | 13 | 8 | 5 |  |
| No | 7 | 5 | 2 |  |
| Clinical stage |  |  |  | 0.284 |
| I-II | 16 | 10 | 6 |  |
| III-IV | 4 | 1 | 3 |  |
